# Transformation Zone Assessment Using Visual Inspection With Acetic Acid Before and After Thermal Ablation: Implications for Follow-Up

**DOI:** 10.1200/GO.22.00241

**Published:** 2023-02-28

**Authors:** Christine Balli, Bruno Kenfack, Apollinaire Horo, Jose Jeronimo, Esther Abatsong, Ania Wisniak, Pierre Vassilakos, Patrick Petignat

**Affiliations:** ^1^ Gynecology Division, Department of Gynecology and Obstetrics, University Hospitals of Geneva, Geneva, Switzerland; ^2^ Department of Biomedical Sciences, University of Dschang, Dschang District Hospital, Dschang, Cameroon; ^3^ Unit of Gynecology and Obstetrics, University Hospital (CHU) of Yopougon, Abidjan, Ivory Coast; ^4^ Division of Cancer Epidemiology and Genetics, Department of Health and Human Services, National Cancer Institute, National Institutes of Health, Bethesda, MD; ^5^ Geneva Foundation for Medical Education and Research, Geneva, Switzerland

## Abstract

Thermal ablation (TA) has become the conventional method for treatment of precancerous cervical lesions in low-resource settings. After TA, both the squamocolumnar junction (SCJ) and the transformation zone (TZ) may be subject to change. Our aim was to evaluate SCJ and TZ variability after TA.

**METHODS:**

Study data were collected in a large prospective trial of a cervical cancer screening campaign in Cameroon. For each patient, two sets of cervical photos (native and with acetic acid) were taken before and 6-12 months after TA. The SCJ and TZ were evaluated independently by three observers according to the WHO nomenclature. When discordances were observed between the type of TZ and SCJ selected by each observer, a corrected TZ was established on the basis of the SCJ categorization. Interobserver agreement for TZ interpretation was evaluated using Cohen's kappa coefficient for agreement between two observers and Fleiss' kappa between three observers.

**RESULTS:**

Fifty consecutive participants were included in the analysis. Seventy-six percent were interpreted as TZ1-2, and 24% as TZ3 before TA. In 56% of cases, the entire SCJ could not be entirely visualized after TA, thus being recategorized as TZ3. Interobserver agreement was fair for diagnosis before TA (Kappa coefficient, 0.34; 95% CI, 0.27 to 0.45) and moderate for diagnosis after TA (Kappa coefficient, 0.40; 95% CI, 0.30 to 0.50). After TA, 36% progressed from TZ1-2 to TZ3, with a moderate interobserver agreement (Kappa coefficient, 0.44; 95% CI, 0.40 to 0.54).

**CONCLUSION:**

We observed a shift of the SCJ into the endocervical canal after TA. A significant proportion of participants had TZ 3 after treatment, raising the question of visual inspection with acetic acid's applicability as a first-line follow-up examination method after TA.

## INTRODUCTION

Sub-Saharan Africa is disproportionately affected by cervical cancer with higher incidence and mortality rates than in any other region of the world.^1,2^ In low-resource settings, cervical cancer incidence and mortality remain high, in part due to various obstacles to implementation of screening programs and treatment of precancer and cancer.^3,4^

**CONTEXT**

**Key Objective**
Does thermal ablation (TA), used in the treatment of precancerous cervical lesions in low- and middle-income countries, change the cervical anatomy?
**Knowledge Generated**
A shift of the transformation zone (TZ) into the endocervical canal 6-12 months after treatment by TA has been observed in 36% of patients. In total, 56% of patients had a non–fully visible TZ (type 3 TZ) after TA, compared with 24% before TA.
**Relevance**
Visual inspection with acetic acid (VIA) is traditionally used for diagnosis and follow-up of precancerous cervical lesions in low- and middle-income countries. A high proportion of women with a type 3 TZ—and thus, out of sight on visual assessment—may limit the value of VIA as a primary follow-up examination after TA. Further studies are needed to determine the diagnostic performance of VIA after TA, and guidelines on the follow-up after TA should be established taking the endocervical shift of the TZ into consideration.

To successfully reduce cervical cancer incidence, effective treatment and appropriate follow-up of women screened positive for precancerous lesions are needed. In high-income countries, Large Loop Excision of the Transformation Zone (LLETZ) and other excisional procedures have become the standard of treatment for women having a cervical intraepithelial neoplasia grade 2 or worse (CIN2+), with a very low rate of persistent disease.^5^ In low-income countries, approaches combining a diagnostic procedure followed by immediate treatment in a screen-and-treat approach using thermal ablation (TA) are recommended by the WHO guidelines.^6,7^ TA is now widely accepted and used in low-resource settings.^8-11^ This procedure destroys abnormal tissue and the transformation zone (TZ) by heating and thus avoids progression to cancer. For patients and health systems, TA is less demanding than LLETZ, with similar efficacy.^12-14^

The WHO has adopted in its guidelines the squamous columnar junction (SCJ) and TZ nomenclature of the International Federation of Cervical Pathology and Colposcopy for visual inspection with acetic acid (VIA) assessment and treatment.^6,15^ Eligibility for ablative treatment following the WHO recommendations requires a fully visible TZ and the whole lesion being completely covered by the probe. Indications for referral to undergo excisional treatment are SCJ and TZ, which are not fully visible (TZ3).^6^ A nonvisible SCJ or a TZ3 constitutes a therapeutic dilemma in low-resource settings, as referral for further investigations (ie, colposcopy, biopsy, and LLETZ) has important consequences for both the patient and the health system, requiring additional time and resources and constituting a risk of loss to follow-up.

Follow-up after TA is important as women who have been treated for CIN2+ have a risk of persistent disease and a higher risk than the general population to develop a new CIN2+ or a cancerous lesion.^16,17^ Post-thermal ablation workup includes a thorough clinical evaluation, clinical history, and VIA including TZ and SCJ assessment. Currently, to the best of our knowledge, no data about the post-thermal ablation prevalence of TZ and SCJ types, nor guidance for the follow-up of women who have been treated, are available. Our aim was to evaluate the change in prevalence of SCJ and TZ types before and after TA.

## METHODS

### Setting and Study Design

The study was nested in a large prospective trial named 3T-study (Test, Triage, and Treat) launched in September 2018 in the Health District of Dschang, in the West Region of Cameroon. The protocol of the 3T-study has been previously described.^18^ Briefly, it is based on human papillomavirus (HPV) primary screening, by self-sampling, followed by triage with VIA using ABCD criteria coupled with digital photos for quality control and supervision.^19-23^ HPV-positive women having a positive VIA are treated in the same visit with TA (if eligible), and women not eligible are referred for further evaluation. Participants are considered eligible for TA if they have a completely visible lesion, which can be fully covered by a flat, endocervical or nipple probe. Inclusion criteria of the 3T-study are women age 30-49 years and willing to participate in the screening program. For the present study, the first 50 participants of the 3T-study having undergone TA were included without further selection. Exclusion criteria were those of the 3T-study (pregnancy and hysterectomy) as well as (1) insufficient quality of images for evaluation and (2) inadequate VIA because of polyps, blood, mucus, or protrusion of the vaginal wall preventing from correctly evaluating the cervix.

### SCJ and TZ Interpretation

**FIG 1:**
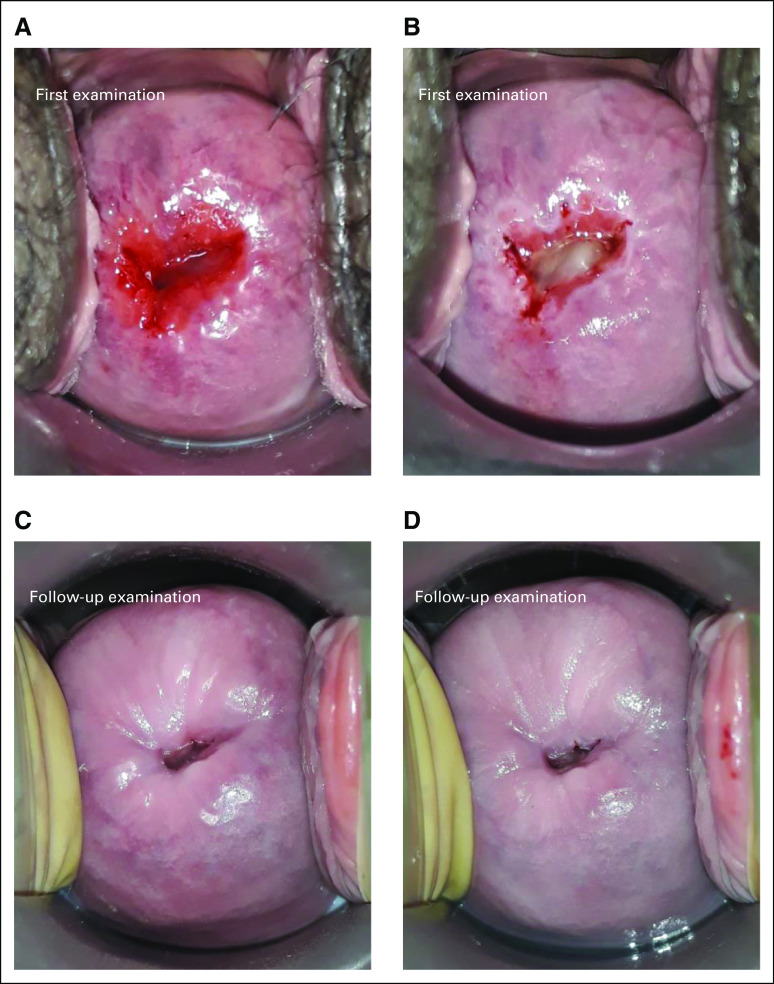
Cervical appearance before and 6-12 months after TA. (A) Cervical appearance before treatment (native). (B) Cervical appearance before treatment after acetic acid application. (C) Cervical appearance 12 months after treatment (native). (D) Twelve months after treatment after acetic acid application. Notably, this situation was interpreted as TZ type 1 at the first visit and TZ type 3 at the follow-up visit after treatment by TA. The new SCJ had moved much closer to the internal OS after treatment. The SCJ is visible as a distinct white line after the application of 5% acetic acid because of the presence of immature squamous metaplastic epithelium adjacent to the new SCJ. SCJ, squamocolumnar junction; TA, thermal ablation; TZ, transformation zone.

The SCJ and TZ types of the 50 selected cases were assessed on digital photos of the cervix (native and with acetic acid) captured at enrollment in the study (prethermal ablation) and at the follow-up visit 6-12 months later (post-thermal ablation; Fig 1). As some participants missed their 6-month follow-up visit or delayed it, post-thermal ablation photos for each participant were selected from the first follow-up visit with images of sufficient quality occurring between 6 and 12 months after enrollment in the study. Images were cropped and introduced in an online Jotform questionnaire^24^ and submitted to the observers for TZ and SCJ interpretation. Classification was performed according to the International Federation of Cervical Pathology and Colposcopy nomenclature, which was coded as the SCJ being visible, partially visible, or not visible and for the TZ as type 1, 2, or 3. Briefly, a type 1 TZ is completely ectocervical, and therefore, the SCJ is fully visible; a type 2 TZ is partially endocervical, but the SCJ is still fully visible; a type 3 TZ extends out of view into the endocervical canal, and the SCJ is not fully visible.^15^ For the interpretation of the present study, we gathered TZ types 1 and 2 in a single category TZ1-2, as they are managed in the same way within the treatment strategy. When discordances were observed between the type of TZ and SCJ selected by each observer, a corrected TZ was established on the basis of the SCJ categorization. Specifically, if the SCJ was described as partially visible or not visible, the corrected TZ was defined as TZ3, whereas a completely visible SCJ was redefined as TZ1-2. The combined interpretation was defined as the type of TZ (original or corrected) and SCJ selected by a majority of observers.

### Observers

Three observers from Cameroon, Ivory Coast, and Peru evaluated the 50 selected cases between January and February 2022. All were experienced in VIA (more than 100 cases/y) and in supervision of cervical cancer screening. Observers were aware that all participants were HPV-positive, interpreted on-site as VIA-positive by first-line health care providers, and considered eligible for TA.

### Image Quality

For each patient, the observers were asked if the image quality was good enough to interpret the SCJ and TZ. It was decided to exclude the image if all three observers considered the quality insufficient.

### Thermal Ablation and Follow-Up

**FIG 2:**
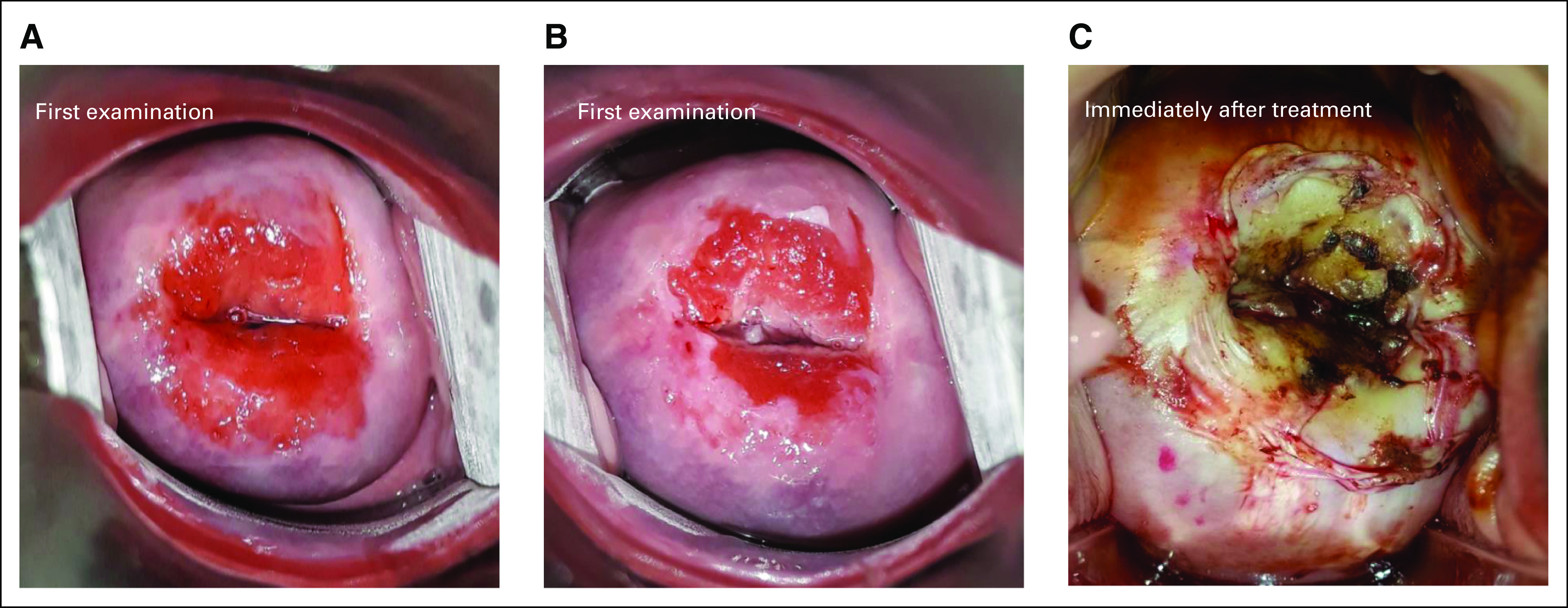
Cervical appearance before and immediately after TA. (A) Cervical appearance before treatment (native). (B) Cervical appearance before treatment after acetic acid application. (C) Cervical appearance immediately after treatment. Notably, after TA, the entire transformation zone is destroyed. TA, thermal ablation.

The TA probe (WISAP; Medical Technology GmbH, Brunnthal/Hofolding, Germany) was heated at 100°C and applied to the cervix for 60 seconds (Fig 2). A two-probe method was routinely used. First, a pointed tip probe was applied onto the cervix and into the endocervical canal, to ensure the entire treatment of the SCJ, followed by a wider flat tip probe, covering the rest of the TZ. The application was repeated until the entire abnormal area and TZ had been covered and treated. All women having undergone TA were invited for a follow-up visit at 6 and 12 months, during which they underwent HPV screening, triage by VIA with smartphone cervical photography, and repeated treatment at 12 months if necessary.

### Ethical Issues

Image acquisition and authorization to use them for research and teaching purposes were obtained from the Cantonal Ethics Board of Geneva, Switzerland (CCER, No. 2017-0110), and the Cameroonian National Ethics Committee for Human Health Research (No. 2018/07/1083/CE/CNERSH/SP). The protocol of the 3T study (the large prospective trial in which this study is nested), was registered under ClinicalTrials.gov (identifier: NCT03757299). All participants provided written informed consent for the acquisition of digital images and their anonymized use for research and training.

### Statistical Analysis

The sample size was calculated using OpenEpi (Version 3) assuming a prevalence of TZ3 of 20% before TA and 50% at the follow-up visit after TA according to our on-site experience. Using Fleiss' chi-squared approximation with continuity correction, a sample of 45 patients was found to be necessary to detect a difference in proportions at the 95% confidence level with an 80% power. We rounded that the number to 50 participants to account for potential missing data.

For sociodemographic characteristics, quantitative variables were expressed as medians and interquartile range (IQR). Categorical variables were expressed as percentages and 95% CIs. The difference in proportions before and after TA was analyzed using Pearson's chi-squared test with a *P* < .05 considered statistically significant. The proportions of participants progressing from TZ1-2 to TZ3 and from TZ3 to TZ1-2 and those with no change in TZ type before and after TA were calculated using both the original and corrected TZ categorizations. Cohen's Kappa was used to compare interobserver variability between two observers, and Fleiss' Kappa for comparisons between three observers. Data were analyzed using the statistical analysis software packages Stata (2019, *Stata Statistical Software: Release 16*; StataCorp, College Station, TX) and R (R Core Team, 2021, *R: A language and environment for statistical computing*; R Foundation for Statistical Computing, Vienna, Austria).

## RESULTS

### Sociodemographic and Clinical Characteristics of Participants

A total of 50 patients were selected. The time between TA and follow-up ranged between 178 and 474 days, with a median of 200 (IQR, 185-239) days. The median age of patients was 36.5 (IQR, 31-42) years (Table 1). Seventy-six percent of patients had a partner, 16% were single, and 8% were divorced or widowed. Six percent were nulliparous, 50% had 1-4 children, and 44% had more than four children, with a median age at first delivery of 21 (IQR, 19-24) years. Fifty-two percent of participants did not use contraception; 22% had an intrauterine device, implant, or injection; 16% used condoms; 6% used a hormonal pill; and 2% had a fallopian tube ligation. In 2% of participants, the contraceptive method was unknown. Only 4% of our study sample were HIV-positive (two participants).

**TABLE 1:**
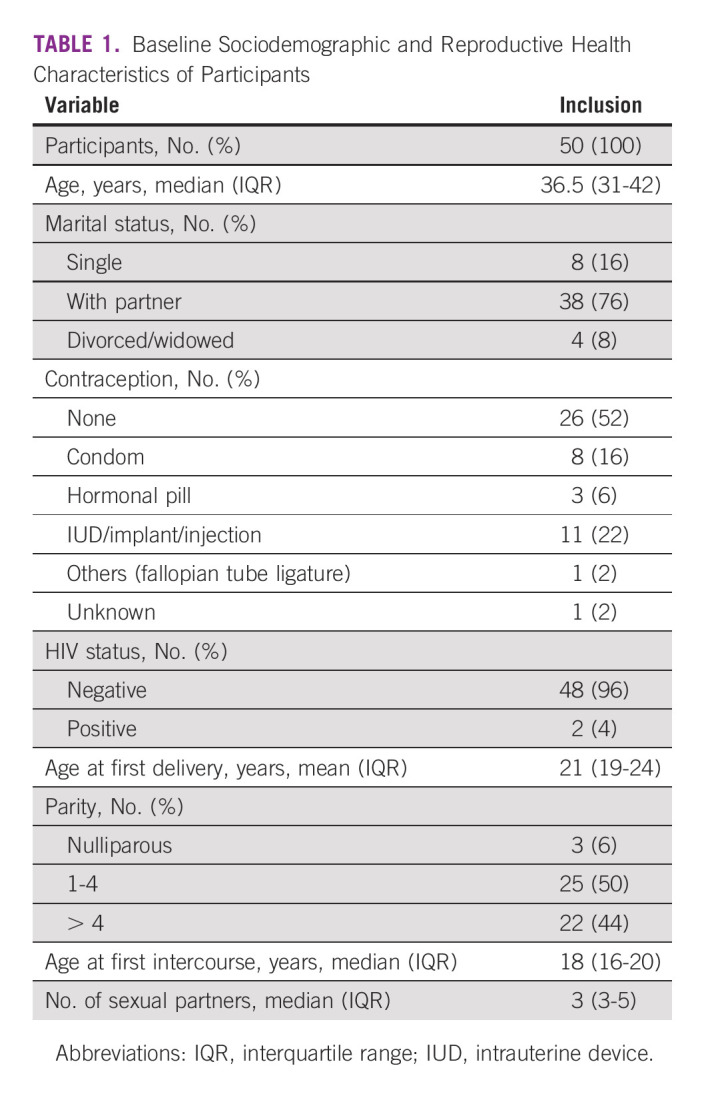
Baseline Sociodemographic and Reproductive Health Characteristics of Participants

### Image Quality

Overall, 50% of photos were considered of good quality by all three observers, 30% by only two of three observers, and 20% only by one of three observers. No image was considered of insufficient quality by all three observers.

### SCJ and TZ Interpretation

Before TA, observer 1 reported 58% completely visible SCJ and 28% and 14% reported partially and not visible SCJ, respectively (Table 2). After TA, this shifted to 16% completely visible SCJ, 54% partially visible SCJ, and 30% not visible SCJ (*P* < .001). Observer 1 reported 88% TZ1-2 and 12% TZ3 before TA and 70% TZ1-2 and 30% TZ3 after TA (*P* = .027). The corrected TZ type (according to SCJ interpretation) for observer 1 was 42% TZ3 before TA versus 84% after TA (*P* < .001). Observer 2 found a higher rate of completely visible SCJ before (82%) and after (58%) TA (*P* = .006) and rates of corrected TZ3 of 18% before and 42% after TA (*P* = .009). Observer 2 noted a similar tendency of progression of the TZ after TA with a 30% shift of corrected TZ1-2 to TZ3. In 3 cases, the corrected TZ for observer 2 went from TZ3 to TZ1-2 after TA. Observer 3 found a less significant change of the TZ and SCJ before and after TA, but still noted a progression of 14% from corrected TZ1-2 to TZ3.

**TABLE 2:**
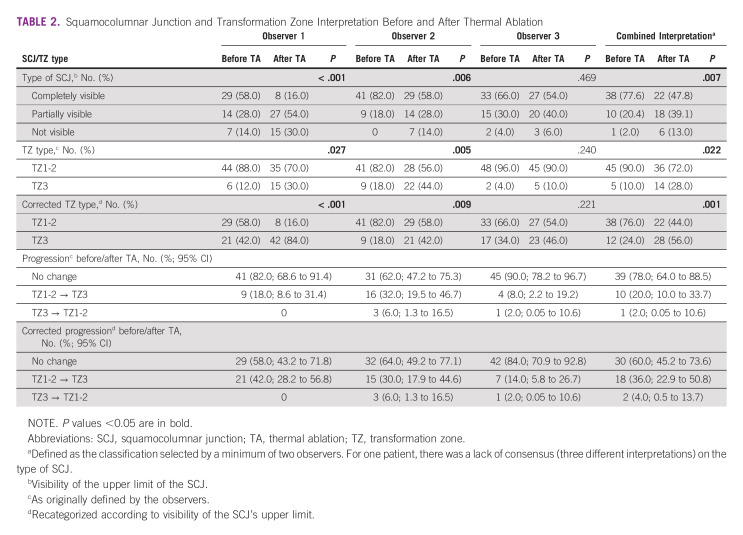
Squamocolumnar Junction and Transformation Zone Interpretation Before and After Thermal Ablation

**FIG 3:**
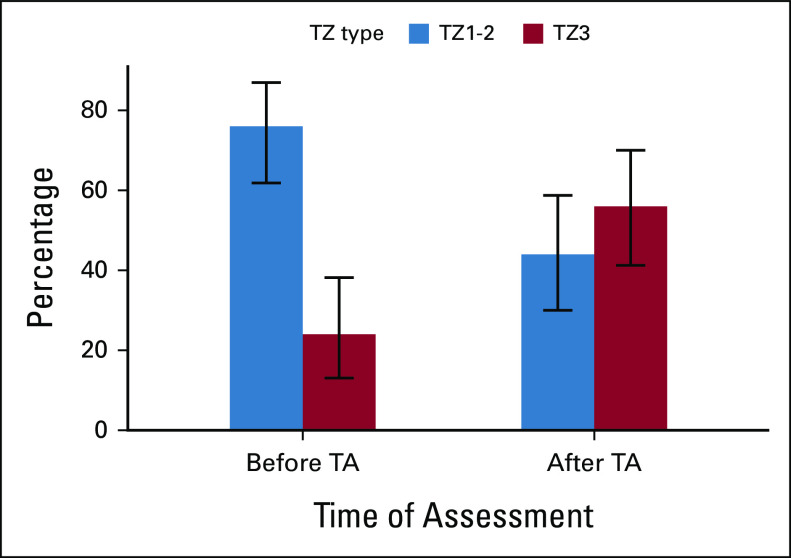
Shift of the TZ type before and after TA. TA, thermal ablation; TZ, transformation zone.

Overall, the combined interpretation showed a prevalence of completely visible SCJ of 77.6% before TA versus 47.8% after TA (*P* = .007). The TZ3 prevalence before TA was 10% versus 28% after TA (*P* = .022). When corrected according to the SCJ interpretation, the TZ3 prevalence was 24% before TA versus 56% after TA (*P* = .001; Fig 3). Progression of TZ1-2 to TZ3 type after TA was 20% according to the combined original interpretations and rose to 36% after TZ correction. In 4% of cases, the TZ type seemed to reverse from TZ3 before TA to TZ1-2 afterward.

### Observer Agreement

Observers 1 and 2 showed substantial agreement (kappa coefficient [K] > 0.60) on the TZ type before and after TA and moderate agreement concerning the progression of TZ type after TA (K = 0.53; Table 3). Observers 1 and 3 showed fair to moderate agreement on the TZ type before and after TA (K = 0.20 and 0.41, respectively) and moderate agreement on the progression of TZ type after TA (K = 0.57). Observers 2 and 3 showed only slight agreement on the TZ type before TA (K = 0.12) and fair agreement on the TZ type after TA (K = 0.25) and on the progression of the TZ after TA (K = 0.31). Overall, the agreement between all three observers was fair for the TZ type before and after TA (K = 0.34 and 0.40, respectively) and moderate concerning the progression of the TZ type after TA (K = 0.44).

**TABLE 3:**
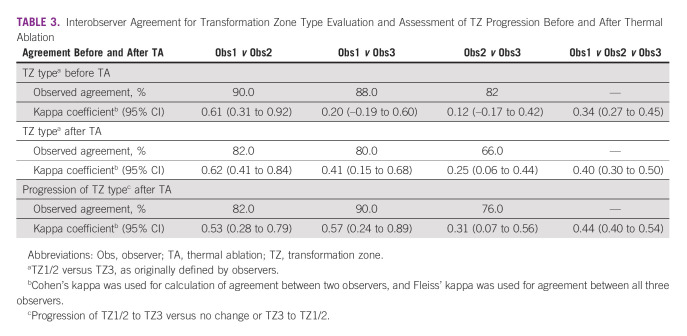
Interobserver Agreement for Transformation Zone Type Evaluation and Assessment of TZ Progression Before and After Thermal Ablation

## DISCUSSION

VIA in the follow-up of patients treated by TA should reliably identify women having a persistent or residual cervical disease that has not been entirely destroyed during treatment or a recurrent disease. This issue may be challenging as residual post-treatment lesions might have characteristics that make them difficult to detect. In our experience, we noticed a great variability of cervical tissue after TA, such as focal thickening, sometimes scarring that may mimic or hide CIN2+ lesions, and a change in the position and visibility of the SCJ and TZ.

In our series, we observed a significant shift of 36% (95% CI, 22.9 to 50.8) from TZ1-2 to TZ3, 6-12 months after TA, with more than half of patients having a TZ3 after treatment (56%, *v* 24% before, *P* = .001). This supports the fact that TA has an impact on the cervical anatomy with a significant change of the SCJ and TZ.

Diagnostic performance of VIA to detect precancerous lesions and cancer is likely to be even lower in women having a TZ3.^25,26^ Thus, the detectability of residual or persistent cervical lesions may decrease as the SCJ becomes less visible after treatment by TA. These issues may make interpretation of VIA more complex during follow-up in a screen-and-treat setting for frontline health care providers. Indeed, current International Agency for Research on Cancer and WHO guidelines stress the importance of using VIA as a screening method and ablation only when the SCJ is fully visible and recommend referring for further evaluation by colposcopy and LLETZ if the TZ is not fully visible.^6^ This may be problematic if adequate access to referral services is not readily available.

We observed that TZ classification was not uniformly used by all observers, with a high heterogeneity of agreement between observers, ranging from substantial (K > 0.61) to slight or fair (K = 0.01-0.20, K = 0.21-0.40). We also observed an intraobserver discordance between the original TZ classification and the reported SCJ visibility, which led us to introduce a corrected TZ, on the basis of SCJ visibility. This has important implications for eligibility for TA, which appears to be highly subjective.

To date, there are no uniform follow-up protocols after TA and the available literature is scarce and frequently overlooks the reliability of VIA after treatment. Traditionally, the same methods that are used for pretreatment diagnosis are also used at a follow-up of 6-12 months after treatment although there is no clear evidence that it increases the detection of persistent or recurrent disease.^27^ In the future and according to the difficulty in the follow-up of patients having a non–fully visible TZ, it is probable that HPV testing (if available) could become the preferred test of cure in low-resource settings. In this context, VIA would be reserved for patients who are still HPV-positive after treatment. If its results are uninterpretable because of the presence of TZ3, we would suggest referral for further investigations.

Alternative methods addressing the limitation of assessment of patients with a non–fully visible TZ are currently being evaluated, such as Artificial Intelligence–based VIA, Artificial Intelligence–based cytology, and HPV rapid testing, which might be promising approaches in the future.

Finally, as TA is associated with a significant change in cervical anatomy, which makes interpretation of VIA more complex, clinicians should avoid overtreatment and optimize their decision strategy to treat only women having a true disease.

The major strength of this study is that patients were their own controls, as VIA findings were compared between pre- and post-thermal ablation images. This minimizes selection bias and other confounding factors. Second, the series are consecutive cases corresponding to a real routine practice, without selection other than for image quality. The main limitation is that we used static images in which the SCJ and TZ may be more difficult to assess, considering that during in-person examination, the provider can improve visualization by manipulating the cervix with a swab or repositioning the speculum. Another limitation is that the SCJ and TZ can change with age, body hormone levels, and menstrual cycle. In this study, we only report the variable of age. Data on menopause status and menstrual cycle were not collected. Nevertheless, heterogeneity of the study sample is limited by an inclusion age of 30-49 years and participants acting as their own controls.

In conclusion, a high number of post-thermal ablation examinations are documented as TZ3, supporting that VIA might have limited value as a primary follow-up examination of patients treated with TA. Further studies need to be conducted to determine the value of VIA after TA to provide better guidance for health care providers.
